# Effect of Catechol Content in Catechol-Conjugated Dextrans on Antiplatelet Performance

**DOI:** 10.3390/polym9080376

**Published:** 2017-08-19

**Authors:** Yeonwoo Jeong, Kwang-A Kim, Sung Min Kang

**Affiliations:** Department of Chemistry, Chungbuk National University, Chungbuk 28644, Korea; ywjeong9104@gmail.com (Y.J.); kwangakim03@gmail.com (K.-A.K.)

**Keywords:** dextran, catechol conjugation, surface coating, antiplatelet

## Abstract

The surface coating of solid substrates using dextrans has gained a great deal of attention, because dextran-coated surfaces show excellent anti-fouling property as well as biocompatibility behavior. Much effort has been made to develop efficient methods for grafting dextrans on solid surfaces. This led to the development of catechol-conjugated dextrans (Dex-C) which can adhere to a number of solid surfaces, inspired by the underwater adhesion behavior of marine mussels. The present study is a systematic investigation of the characteristics of surface coatings developed with Dex-C. Various Dex-C with different catechol contents were synthesized and used as a surface coating material. The effect of catechol content on surface coating and antiplatelet performance was investigated.

## 1. Introduction

Dextran (Dex) is a polysaccharide consisting of glucose molecules, and has been of importance due to its use in medical applications as an antiplatelet and blood volume expander [1]. Particularly, the antiplatelet properties of Dex have been used in combination with several surface coating techniques with the aim of preparing blood-compatible medical devices [2,3,4,5,6,7,8,9,10,11]. Previous studies have revealed that Dex-coated surfaces could effectively limit cell adhesion as well as protein adsorption [4,5,7,8]. For example, Massia et al. reported the covalent immobilization of Dex on a glass surface [5]. In the study, amino groups were firstly introduced on the glass surface by using 3-aminopropryltriethoxysilane, an organosilane coupling agent. Activated Dex, which is oxidized by sodium periodate, was then immobilized on the glass surface via reductive amination between amino groups of the glass surface and aldehyde groups of the activated Dex. Fibroblast cell adhesion and spreading was effectively inhibited on the Dex-immobilized glass surfaces, as opposed to untreated surfaces.

Although the covalent methods of Dex grafting on solid surfaces have been successfully employed, those methods have drawbacks, in that complicated steps and harsh reaction conditions are required. Therefore, the development of advanced grafting methods is needed. Recently, a mussel-inspired approach in which Dex can be easily grafted on solid substrates was investigated [9,10,11]. Given that the catechol is known to play an important role in the strong underwater adhesion of marine mussels, catechol-conjugated polymers were developed for use as functional coatings of diverse substrates [12,13,14]. In the case of Dex, Park et al. synthesized catechol-conjugated Dex (Dex-C) and applied it onto titanium (Ti) surfaces; Dex-C with a catechol grafting density of ~7% was prepared by a carbamate-bond forming reaction, and employed as in surface coating of Ti in anti-fouling applications [9,10]. Dex-C-coated Ti surfaces exhibited excellent resistance against protein and cell adhesions. Despite the aforementioned surface coating ability and anti-fouling properties of Dex-C, there are areas of fundamental research that have not yet been fully investigated, such as the effect of catechol content in Dex-C on surface coating and anti-fouling properties. In this respect, herein, Dex-C with various catechol contents were synthesized for coating Ti surfaces. The surface coating efficiency as a function of catechol content was investigated by spectroscopic ellipsometry, contact angle goniometry, and X-ray photoelectron spectroscopy (XPS). The anti-fouling properties of Dex-C-coated surfaces were evaluated by measuring the platelet density attached to surfaces.

## 2. Experimental Section

### 2.1. Materials

Dextran (Dex, 6 k MW, from *Leuconostoc* spp., Sigma-Aldrich, St. Louis, MO, USA ), dopamine hydrochloride (98%, Sigma-Aldrich), Trizma base (99%, Sigma), Trizma·HCl (99%, Sigma), 1,1’-carbonyldiimidazole (CDI, Sigma-Aldrich), hydrochloric acid (HCl, 35~37%, Duksan, Ansan, Korea), absolute ethanol (Merck, Kenilworth, NJ, USA), dimethyl sulfoxide (DMSO, 99%, TCI, Tokyo, Japan), and acetone (99%, Daejung Chemicals & Metal, Shiheung, Korea) were used as received. A concentrated platelet solution (1.04 × 10^6^ cells/mL) was obtained from Red Cross blood center (Chungbuk, Korea), and the platelets were used as received. The use of human platelets for this study was approved by the institutional review board (IRB) of Chungbuk National University.

### 2.2. Synthesis of Catechol-Conjugated Dextrans (Dex-C)

Dex-C was synthesized according to the previous report [9]. Dex (162.1 mg) was dissolved in 5 mL of DMSO at room temperature. CDI (324.3 mg) in 2 mL of DMSO was added to the Dex solution, and the resulting solution was stirred at room temperature for 30 min. Dopamine hydrochloride (189.6 mg) which was dissolved in 1 mL of DMSO was then added to the CDI-activated Dex solution. The conjugation reaction between CDI-activated Dex and dopamine hydrochloride was carried out at room temperature. After overnight reaction, 30 mL of deionized (DI) water was added to the solution. Subsequently, precipitates formed in the solution were removed, and the water-soluble part was transferred to a dialysis membrane (MWCO = 3500) and dialyzed for 24 h to remove unreacted coupling reagents. Acidified water obtained by adding 1 mL of 5 M HCl to 1 L of DI water was used for a dialysis procedure. This process was repeated with different molar ratios of reactants (monomer unit of Dex:dopamine:CDI = 9:1:1.5, 9:1:6, 1:1:1, and 1:1:2). The final products were freeze-dried and stored in a refrigerator before use.

### 2.3. Dex-C Coating on Solid Substrates

Ti/TiO_2_ substrates were prepared by thermal evaporation of 100 nm of Ti onto silicon wafers. Ti/TiO_2_ substrates (1 cm × 1 cm) were cleaned with acetone or ethanol by sonication prior to use. Dex-C coating was carried out by immersing substrates in a buffered solution (5 mg of Dex-C per 1 mL of 50 mM Tris, pH 8.5) at room temperature for 24 h. The coated substrates were rinsed with DI water and blow-dried under a steam of nitrogen gas.

### 2.4. Platelet Adhesion

Uncoated and Dex-C-coated Ti/TiO_2_ substrates were incubated in 0.5 mL of platelet media (1.04 × 10^6^ cells/mL) for 24 h. After incubation at room temperature [15], substrates were rinsed by phosphate-buffered saline (PBS, Sigma, St. Louis, MO, USA) solution (pH 7.4) and immersed into a glutaraldehyde solution (2.5%) for 24 h. After that, substrates were dehydrated by immersing into a series of ethanol solutions (25%, 50%, 75%, 95%, and 100%). The attached platelets were characterized by field emission SEM (FE-SEM).

### 2.5. Characterizations

XPS was carried out using a PHI Quantera II (ULVAC-PHI, Inc., Chigasaki, Japan) with an Al Kα X-ray source and ultrahigh vacuum (~10^−10^ mbar). The thickness of the organic layers on solid substrates was measured using a spectroscopic ellipsometer (Elli-SE, Ellipso Technology, Suwon, Korea). Static water contact angle measurements were carried out using a Phoenix-300 TOUCH goniometer (Surface Electro Optics Co., Ltd., Suwon, Korea). UV–Vis spectra of products (0.25 mg/mL) were obtained using a UV–Vis spectrophotometer (Libra S70, Biochrom, UK). Fourier transform infrared (FT-IR) spectra were acquired using an ALPHA FT-IR spectrometer (Bruker, Germany). FE-SEM imaging was performed by an Ultra Plus microscope (Zeiss, Germany) with an accelerating voltage of 3 kV, after sputter-coating with platinum.

## 3. Results and Discussion

**Figure 1:**
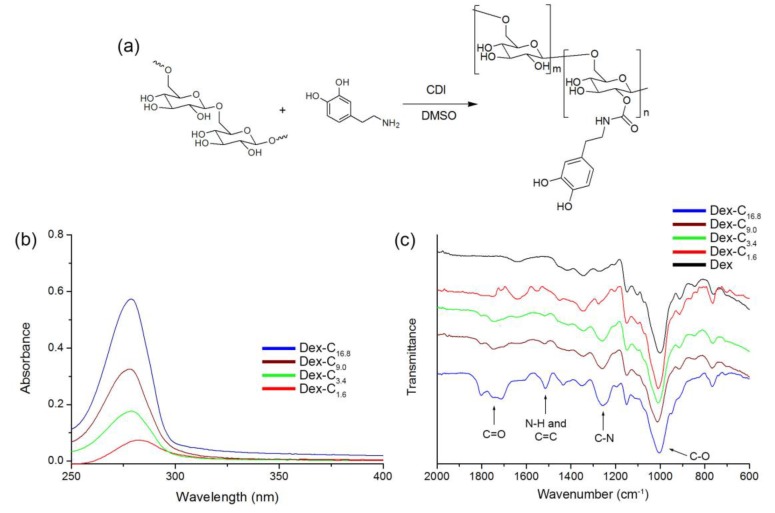
(**a**) Synthesis of catechol-conjugated dextran (Dex-C); (**b**) UV–Vis; and (**c**) Fourier transform infrared (FT-IR) spectra of Dex, Dex-C_1.6_, Dex-C_3.4_, Dex-C_9.0_, and Dex-C_16.8_.

Dex-C was synthesized via the carbamate bond forming reaction (Figure 1a) [9]. The hydroxyl groups of Dex were activated by using carbonyldiimidazole (CDI) and reacted with dopamine, which is a catecholamine. In order to synthesize Dex-C with various catechol contents, the molar ratio of reactants was varied accordingly (see the Experimental section). The conjugation of Dex with dopamine was analyzed by UV–Vis and FT-IR spectroscopy. Specifically, UV–Vis spectra of products revealed that dopamine was successfully conjugated to Dex, as evidenced by the presence of a peak at 280 nm, corresponding to the catechol of dopamine (Figure 1b) [9]. The absorbance at 280 nm was also used to quantify the extent of catechol conjugation with the hydroxyl groups of Dex. The calibration curve was generated using known concentrations of five dopamine solutions (Figure S1, Supplementary Material). The catechol content in resulting polymers was calculated by comparing with a calibration curve, and the grafting densities of catechol to glucose (the repeating unit of Dex) were found to be 1.6, 3.4, 9.0, and 16.8 mol %. The final polymers were denoted as Dex-C_1.6_, Dex-C_3.4_, Dex-C_9.0_, and Dex-C_16.8_. The synthesis of Dex-C was further analyzed by FT-IR spectroscopy. Unlike the Dex, Dex-C showed new peaks including C=O stretching (1806 and 1740 cm^−1^) and N–H bending (1510 cm^−1^) (Figure 1c). With an increase in catechol content in Dex-C, an increase in intensities of these characteristic peaks was also observed. Overall, these results indicated that Dex-C with various catechol contents were successfully prepared by a simple chemical reaction.

**Figure 2:**
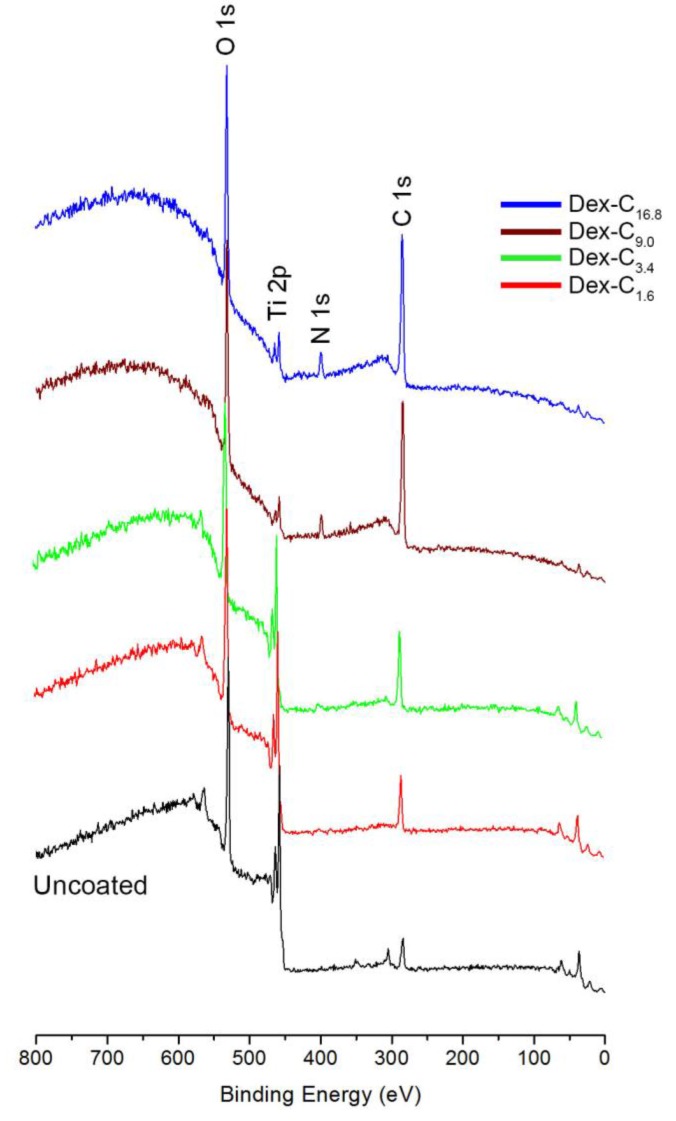
X-ray photoelectron spectra of uncoated, Dex-C_1.6_, Dex-C_3.4_, Dex-C_9.0_, and Dex-C_16.8_-coated Ti/TiO_2_ surfaces.

Synthesized Dex-C were subsequently used for the surface coating of solid substrates. Ti/TiO_2_ was chosen as a model substrate, because Ti-based substrates are widely used in biomedical devices where the control of unnecessary biofouling is crucial [16]. Moreover, it is practically advantageous to use Ti/TiO_2_, since it is compatible with conventional surface characterization techniques. After a 24-h immersion of Ti/TiO_2_ substrates into four types of Dex-C solutions, surfaces were characterized by XPS, spectroscopic ellipsometry, and contact angle goniometry. According to the XPS analysis, all Ti/TiO_2_ surfaces coated by Dex-C (regardless of grafting density) showed C 1s, N 1s, O 1s, and Ti 2p peaks, of which C 1s, N 1s, and O 1s peaks originated from Dex-C (Figure 2). Quantitative analysis of the surface chemical composition of surfaces was also performed in order to assess the effect of catechol content on surface coating efficiency. It was anticipated that highly more efficient surface coatings would be attained when using the Dex-C with higher catechol content. This is associated with the positive effect of catechols on the performance of surface coatings [17].

As shown in Table 1, increased intensity of the C 1s and N 1s peaks was observed in the case of Dex-C. Decreased intensity of Ti 2p peaks was observed with increasing catechol content in the Dex-C being used in surface coating. Especially given that the intensity of peaks of the underlying substrate is sensitive to the amount of upper layers (i.e. Dex-C layer), the sequential decrease of Ti 2p peaks was direct evidence of enhanced coating efficiency when the catechol content in Dex-C was increased. The analysis of the areal ratio between O 1s and Ti 2p also gave us useful information to investigate the coating efficiency by Dex-C. The ratio (O 1s/Ti 2p) of uncoated Ti/TiO_2_ surfaces was 2.34, and increased to 3.43, 4.67, 5.43, and 19.2 after Dex-C_1.6_, Dex-C_3.4_, Dex-C_9.0_, and Dex-C_16.8_ coatings, respectively. This implies that the contribution of Ti/TiO_2_ surface for O 1s peak decreases by Dex-C coating, in which the coating efficiency is enhanced as the catechol content in Dex-C increases. Spectroscopic ellipsometry results also suggested that the surface coating efficiency is increased by increasing the catechol content; 0.9, 1.6, 3.8, and 5.3 nm-thick Dex-C layers were deposited on Ti/TiO_2_ surfaces by Dex-C_1.6_, Dex-C_3.4_, Dex-C_9.0_, and Dex-C_16.8_ coatings, respectively (Table 1, Figure S2, Supplementary Material). The stability of the Dex-C coating on solid substrates is critical for practical applications. The mechanical stability of the Dex-C coating was examined by measuring the thickness change upon strong ultrasonication (40 kHz, 75 W). Dex-C_16.8_-coated surfaces were used in this study, and the coating layer remained stable even after ultrasonication for 30 min, indicating the robustness of the Dex-C coating (Figure S3, Supplementary Material).

**Figure 3:**
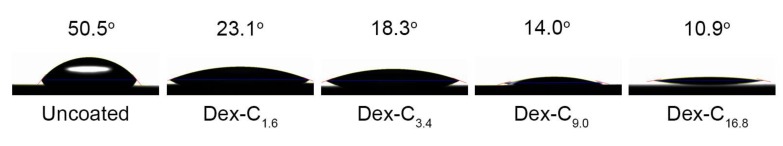
Water contact angle images of uncoated, Dex-C_1.6_, Dex-C_3.4_, Dex-C_9.0_, and Dex-C_16.8_-coated Ti/TiO_2_ surfaces.

Prior to the use of Dex-C-coated Ti/TiO_2_ surfaces in potential anti-fouling applications, changes in surface wettability were assessed, as this is highly related to the overall anti-fouling performance [18]. Changes in water contact angle of surfaces were comparable with results obtained by XPS and ellipsometry. The Dex-C-coated Ti/TiO_2_ surfaces became more hydrophilic than uncoated Ti/TiO_2_; Dex-C_16.8_-coated surfaces exhibited the highest hydrophilicity (Figure 3). This suggests that the increase in coating thickness by Dex-C_16.8_ provided complete coverage of Ti/TiO_2_ surfaces, resulting in enhanced wettability. After confirming that surface wettability of Ti/TiO_2_ substrates can be tailored by using different Dex-C in surface coatings, anti-fouling assays were conducted.

**Figure 4:**
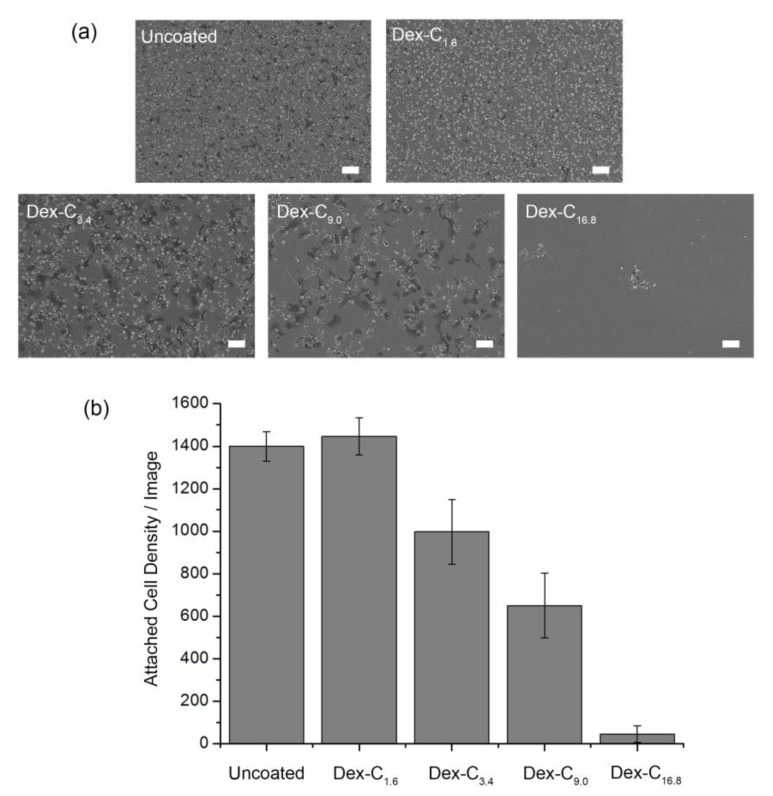
(**a**) SEM images and (**b**) quantification of platelets attached to uncoated, Dex-C_1.6_, Dex-C_3.4_, Dex-C_9.0_, and Dex-C_16.8_-coated Ti/TiO_2_ surfaces. All scale bars are 10 μm. Each point indicates the mean from 15 counts from three replicate samples, and the error bars display 95% confidence limits.

Platelets were used as a model foulant, given their adverse effects on the use of blood-contacting medical implants. Given that dextran can inhibit cell adhesion on surfaces, and the fact that the antifouling performance is strongly related with surface hydrophilicity, the Dex-C_16.8_ coating was expected to show superior antiplatelet properties. Platelets were seeded onto each sample and incubated for 24 h. Fouling behavior of platelets on surfaces was investigated by scanning electron microscopy (SEM). As shown in Figure 4, fouling behavior of platelets on Dex-C-coated surfaces significantly varied depending on the catechol content in Dex-C. The attached platelet density was calculated to be 1446, 997, 650, and 45 cells/image on the Dex-C_1.6_-, Dex-C_3.4_-, Dex-C_9.0_-, and Dex-C_16.8_-coated Ti/TiO_2_ surfaces, respectively, whereas 1399 cells were attached to the uncoated Ti/TiO_2_ surfaces. Morphological changes (spreading and pseudopodia emission) that indicate the activation of the platelets were also observed in the SEM analysis (Figure S4, Supplementary Material). Overall, Dex-C coatings conferred anti-fouling properties to the surfaces, with the only exception being Dex-C_1.6_. The platelet adhesion was reduced by 28.7%, 53.5%, and 96.8% after surface coating with Dex-C_3.4_, Dex-C_9.0_, and Dex-C_16.8_, respectively. The results suggest that both coating thickness and anti-fouling performance can be enhanced by increasing the catechol content in Dex-C. Analyzing with the results from other groups, the antiplatelet performance of the Dex-C_16.8_ coating was comparable to that achieved with PEG coatings, but was weaker than that achieved with zwitterionic polymer coatings; it was reported that sulfobetaine and carboxybetaine polymer coatings reduce platelet adhesion by ~98%, whereas PEG coatings reduce the adhesion by ~95% [19,20]. Although the interaction between Dex-C and platelet is not fully understood, the anti-fouling property can be attributed to the formation of hydration layers interrupting direct contact of platelets with the surfaces [18]. Given that catechols play an important role in immobilizing polymers on surfaces as well as in intermolecular crosslinking of polymers [21,22], increase of catechol content in Dex-C can enable thicker Dex-C coating and the application of denser hydration layers on surfaces. Therefore, the Dex-C_16.8_ coating exhibited the highest anti-fouling performance.

## 4. Conclusions

In summary, catechol-conjugated dextrans (Dex-C) with different catechol content were synthesized in order to investigate the effect of catechol content on surface coating efficiency. Surface coating was carried out by immersing solid substrates into solutions of Dex-C with different grafting densities (1.6, 3.4, 9.0, and 16.8 mol %). After careful assessment of Dex-C-coated surfaces, the platelet adhesion behavior on surfaces was investigated. Increasing the catechol content in Dex-C resulted in enhanced surface coating and antiplatelet properties. In this study, the optimum grafting density of catechol to glucose was 16.8 mol %. It is hence concluded that optimization of the chemical composition of Dex-C is an area of research that merits attention, given that precise control of surface coatings can be achieved.

## Figures and Tables

**Table 1:** Atomic composition (%) of uncoated, Dex-C_1.6_, Dex-C_3.4_, Dex-C_9.0_, and Dex-C_16.8_-coated Ti/TiO_2_ surfaces. Thicknesses of Dex-C layers on Ti/TiO_2_ surfaces after Dex-C_1.6_, Dex-C_3.4_, Dex-C_9.0_, and Dex-C_16.8_ coatings.

	C 1s	N 1s	O 1s	Ti 2p	O 1s/Ti 2p	Thickness (nm)
Uncoated	28.6	1.2	49.2	21.0	2.34	-
Dex-C_1.6_	31.4	2.6	51.1	14.9	3.43	0.9
Dex-C_3.4_	40.0	2.1	47.7	10.2	4.67	1.6
Dex-C_9.0_	41.8	2.3	47.2	8.7	5.43	3.8
Dex-C_16.8_	57.5	4.0	36.6	1.9	19.2	5.3

## Supplementary Materials

Supplementary Materials are available online at www.mdpi.com/2073-4360/9/8/376/s1, Figure S1: The calibration curve of dopamine, Figure S2: Spectroscopic ellipsometry data of organic layers on Ti/TiO_2_ surfaces after Dex-C_1.6_, Dex-C_3.4_, Dex-C_9.0_, and Dex-C_16.8_ coatings (solid line: model, dotted line: experimental data), Figure S3: Thicknesses of remaining Dex-C_16.8_ on Ti/TiO_2_ surfaces after sonication for 5, 10, and 30 min. The thickness of Dex-C_16.8_ on Ti/TiO_2_ surfaces before sonication was taken as 1. Error bars represent the standard deviation, Figure S4: Magnified SEM images of platelets attached to uncoated, Dex-C_1.6_, Dex-C_3.4_, Dex-C_9.0_, and Dex-C_16.8_-coated Ti/TiO_2_ surfaces. All scale bars are 2 μm.
