# Supplementary material for: Effect of Catechol Content in Catechol-Conjugated Dextrans on Antiplatelet Performance
Source: Polymers (Basel). 2017 Aug 19;9(8):376. doi: 10.3390/polym9080376 (PMC6418717; doi:10.3390/polym9080376)
Supplement: Supplementary file 1 [file polymers-09-00376-s001.pdf]

polymers

Supplementary Materials: Effect of Catechol Content in Catechol-Conjugated Dextrans on Antiplatelet Performance

Yeonwoo Jeong [1,†], Kwang-A Kim [1,†] and Sung Min Kang [1,*]

[1] Department of Chemistry, Chungbuk National University, Chungbuk 28644, Republic of Korea; ywjeong9104@gmail.com (Y.J); kwangakim03@gmail.com (K.-A.K)
* Correspondence: smk16@chungbuk.ac.kr; Tel.: +82-43-261-2289
† These authors equally contributed to this work.

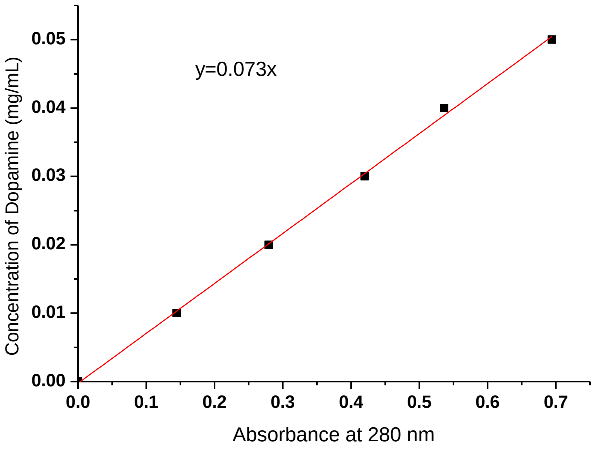

Figure S1. The calibration curve of dopamine.

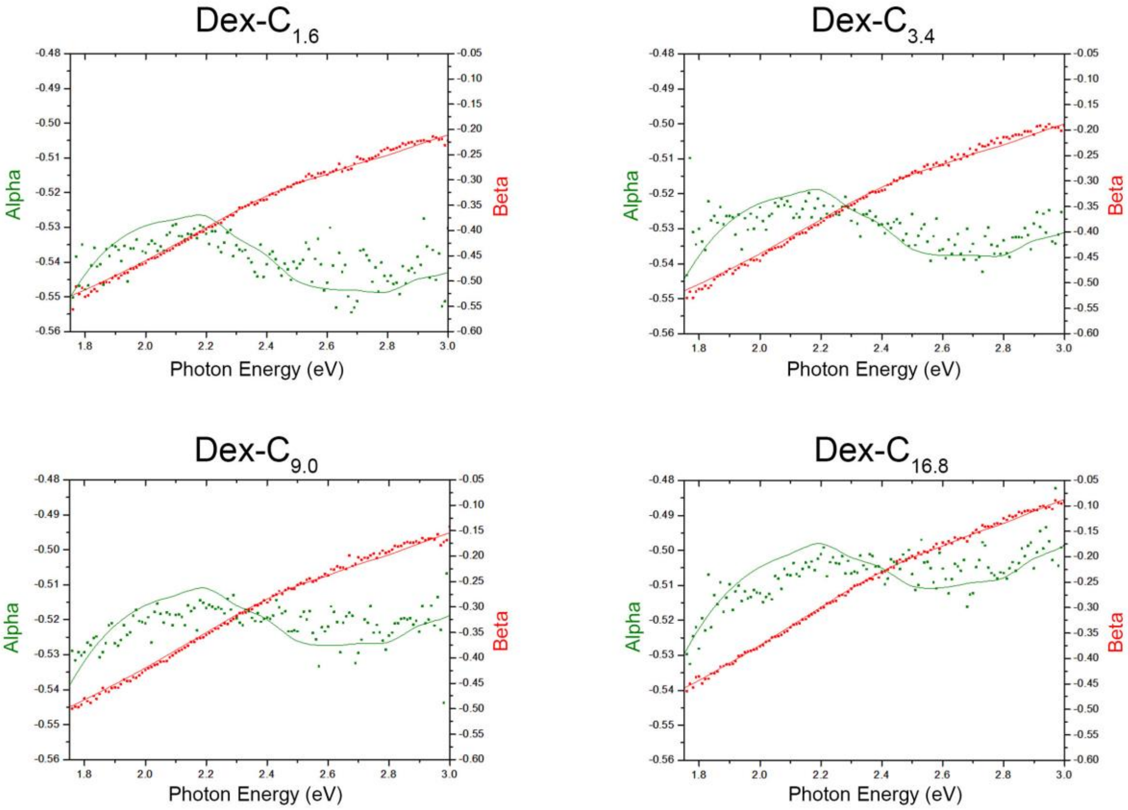

Figure S2. Spectroscopic ellipsometry data of organic layers on Ti/TiO_2 surfaces after Dex-$C_{1.6}$, Dex-$C_{3.4}$, Dex-$C_{9.0}$, and Dex-$C_{16.8}$ coatings (solid line: model, dotted line: experimental data).

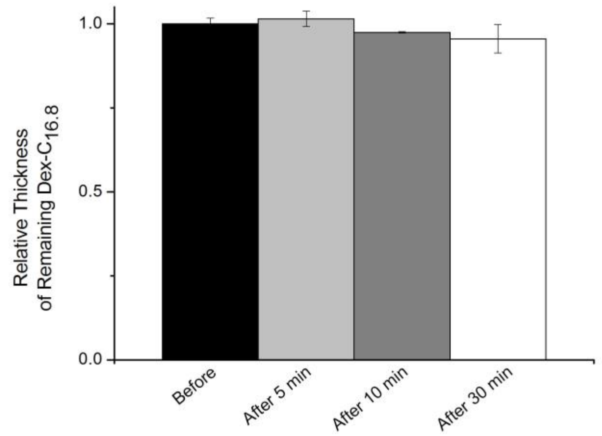

Figure S3. Thicknesses of remaining Dex-$C_{16.8}$ on Ti/TiO_2 surfaces after sonication for 5, 10, and 30 min. The thickness of Dex-$C_{16.8}$ on Ti/TiO_2 surfaces before sonication was taken as 1. Error bars represent the standard deviation.

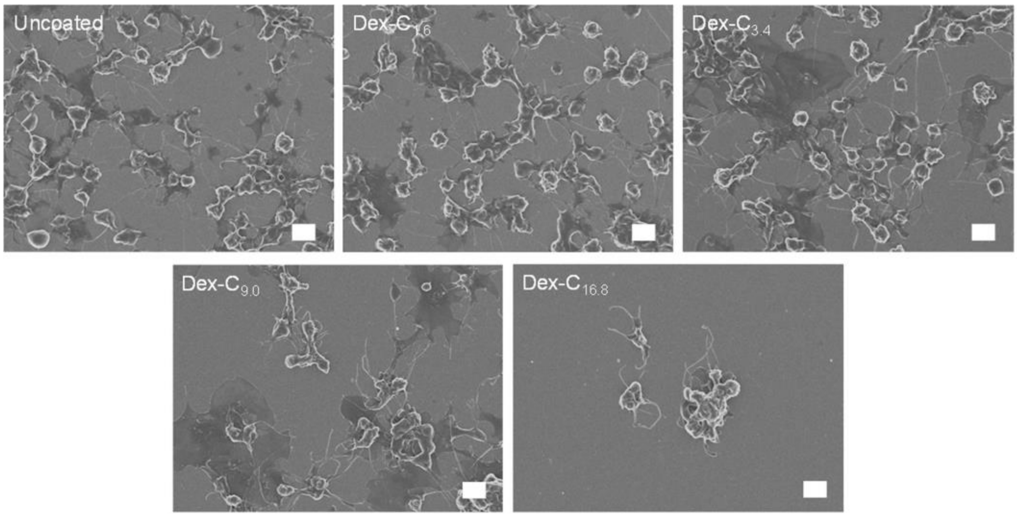

Figure S4. Magnified SEM images of platelets attached to uncoated, Dex-$C_{1.6}$, Dex-$C_{3.4}$, Dex-$C_{9.0}$, and Dex-$C_{16.8}$-coated Ti/TiO_2 surfaces. All scale bars are 2 μm.
